# The Understanding and Interpretation of Innovative Technology-Enabled Multidimensional Physical Activity Feedback in Patients at Risk of Future Chronic Disease

**DOI:** 10.1371/journal.pone.0126156

**Published:** 2015-05-04

**Authors:** Max J. Western, Oliver J. Peacock, Afroditi Stathi, Dylan Thompson

**Affiliations:** Department for Health, University of Bath, Bath, BA2 7AY, United Kingdom; Vanderbilt University, UNITED STATES

## Abstract

**Background:**

Innovative physical activity monitoring technology can be used to depict rich visual feedback that encompasses the various aspects of physical activity known to be important for health. However, it is unknown whether patients who are at risk of chronic disease would understand such sophisticated personalised feedback or whether they would find it useful and motivating. The purpose of the present study was to determine whether technology-enabled multidimensional physical activity graphics and visualisations are comprehensible and usable for patients at risk of chronic disease.

**Method:**

We developed several iterations of graphics depicting minute-by-minute activity patterns and integrated physical activity health targets. Subsequently, patients at moderate/high risk of chronic disease (n=29) and healthcare practitioners (n=15) from South West England underwent full 7-days activity monitoring followed by individual semi-structured interviews in which they were asked to comment on their own personalised visual feedback Framework analysis was used to gauge their interpretation and of personalised feedback, graphics and visualisations.

**Results:**

We identified two main components focussing on (a) the interpretation of feedback designs and data and (b) the impact of personalised visual physical activity feedback on facilitation of health behaviour change. Participants demonstrated a clear ability to understand the sophisticated personal information plus an enhanced physical activity knowledge. They reported that receiving multidimensional feedback was motivating and could be usefully applied to facilitate their efforts in becoming more physically active.

**Conclusion:**

Multidimensional physical activity feedback can be made comprehensible, informative and motivational by using appropriate graphics and visualisations. There is an opportunity to exploit the full potential created by technological innovation and provide sophisticated personalised physical activity feedback as an adjunct to support behaviour change.

## Background

Physical inactivity has a powerful effect on global health and an increase in activity would have an enormous impact on the burden of chronic disease [1]. Of all the strategies implemented to positively change an individual’s behaviour, self-monitoring is one of the most effective [2,3]. In the past few years, technological innovation has transformed the landscape and a plethora of instruments are now commercially available for the self-monitoring of physical activity. These include devices produced by major international companies such as Fitbit, Jawbone UP, GENEActive, Philips DirectLife and Nike+ Fuelband. Large manufacturers such as Samsung and Apple are reportedly about to enter the market [4]. Some of these devices have only limited published validity to date but it is noteworthy that one commercially available multi-sensor instrument from Bodymedia is already classified by the US Food and Drug Administration (FDA) as a Class II medical device. Thus, as instruments become more accurate, affordable, comfortable and discrete [5] millions of people around the world are beginning to use physical activity monitoring technologies and such self-monitoring will become increasingly common in the future.

We recently demonstrated that using the data collected from even the most sophisticated physical activity monitors provides erroneous information about an individual’s physical activity unless this includes a multidimensional profile constructed across the key physical activity dimensions [6]. It is quite possible for a given person to score highly in one physical activity dimension but low in another (e.g. one could engage in substantial vigorous intensity activity but still spend over 80% of their day sedentary) [6]. This is a problem because people sometimes focus on just certain physical activity behaviours without taking into account other dimensions and this could lead to misguided perceptions and expectations. For example, an individual with a weight-loss goal who substantially increases their vigorous intensity structured physical activity might only see a relatively modest impact on overall energy expenditure [7]. Knowledge of all the important physical activity dimensions would remove the potential ambiguity in understanding how their behaviour relates to their goals as well as providing more behavioural options that align to their needs and preferences and offer sustainable solutions [8].

Although we now have the technology to provide feedback that integrates the important multidimensional health-harnessing aspects of physical activity this potentially introduces new risks and challenges. An understanding of personal physical activity is integral to various models of behaviour change and regulation [9,10]. In this context, sophisticated multidimensional physical activity feedback could be seen as more confusing and/or difficult to interpret than simple unidimensional messages. Before we can capitalise on technological innovation, it is important to establish that people can understand multidimensional physical activity feedback in terms of what the feedback represents, the concept of different physical activity dimensions, and the overall meaning of personalised data [8]. There is good evidence that people and patients prefer visual and meaningful images rather than numerical scores and these can be used to increase attention and comprehension of health education information [11,12]. Clearly, the design of the graphical images and representation of multidimensional physical activity feedback will be important for optimising its usefulness as a tool for behaviour change.

To date, there has been very little attempt to determine whether people can understand the information that is available and provided with the advent of increasingly sophisticated physical activity monitors. In particular, there has been no attempt to establish that people can handle potentially complex and conflicting information across the biologically healthful physical activity dimensions. This is especially important in clinical populations who would benefit most from a change in physical activity behaviour (e.g., as a route to manage their risk of chronic disease) [13]. Thus, the purpose of this study is two-fold (i) to develop innovative ways to present multidimensional and sophisticated physical activity feedback to enable self-monitoring and (ii) to explore the understanding, interpretation and potential utility of personalised physical activity feedback amongst patients at future risk of chronic disease and corresponding healthcare practitioners.

## Methods

### Experimental design

We worked with professional infographics specialists to develop multidimensional physical activity visualisations and then evaluated whether patients and healthcare professionals could comprehend these designs and personal feedback on their physical activity and whether they subsequently found this information useful.

### Ethics Statement

Ethical approval for the study was obtained from the National Research Ethics Service Committee South West (REC reference 12/SW/0374).

### Multidimensional visualisations

**Fig 1:**
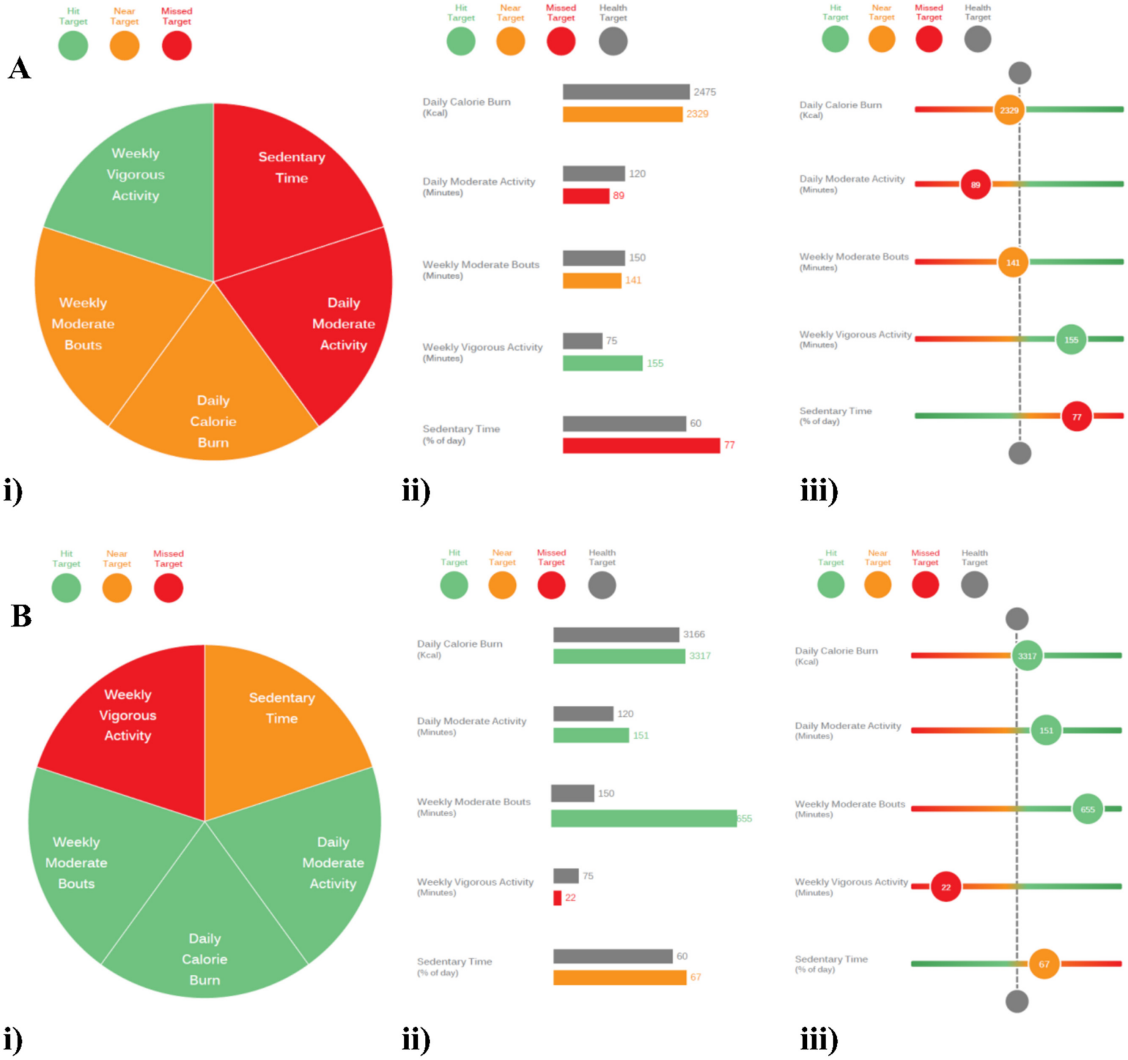
Two examples of the 3 variants of infographics depicting the multidimensional physical activity behavioural recommendations. Green represents a ‘hit’ target, amber a ‘near’ target (within 25%) and red a ‘missed’ target (>25% away). Graphic i) is a simple colour coded wheel format where each segment represents each dimension but has no magnitude; ii) uses a reference target bar to compare a coloured bar scaled to the relative value attained within each dimension; and graphic iii) places the individuals performance for each guideline as a bubble on a sliding scale relative to the target value represented by the central line. The varied nature of physical activity ‘status’ is highlighted by the data from the two participants where A is an individual who has hit their vigorous activity target and is short on the other four dimensions and B is a participant who has a high PAL and considerable moderate intensity activity but is still quite sedentary and has very little vigorous intensity activity.

The infographics we used to depict the physical activity data were created in collaboration with Information is Beautiful and aligned to a design process model [14]. An iterative process was used to develop three sections of information: activity patterns over a day or week, summary graphics of time and energy spent in varying activity intensities, and depictions of performance in relation to multidimensional health targets. Following a phase of piloting and refining initial designs with health professionals (n = 2) and members of the general public (n = 2), a final booklet containing three distinct visualisations for each section of information was developed and shown to participants at interview with their personalised data (an example of this booklet for one participant can be found in S1 Fig). Fig 1 provides two extracts and examples of the multidimensional physical activity profiles.

### Participants

Patients (n = 30) from two general practices were invited to take part if they had been identified as being at moderate (10–19.9%) or high (>20%) risk of cardiovascular disease and/or type 2 diabetes (http://qintervention.org/). Purposive sampling was used to recruit 15 healthcare professionals (HCPs) including 3 general practitioners, 3 nurses/healthcare assistants, 3 research nurses, and 6 physical activity healthcare trainers from two regions in the UK (Bath and North East Somerset and Wiltshire). HCPs were included because of their unique understanding developed over years of working with a wide range of patients. All participants provided written informed consent.

### Procedure

Participants were provided with an arm-mounted Bodymedia Armband (SenseWear Pro 8.0, Pittsburgh, USA), which accurately estimates energy expenditure [15–17]. Participants were instructed to wear the device for seven consecutive days commencing at midnight and asked to only remove the device for showering or water-based activities [18]. Minutes spent in the distinct intensity thresholds based on metabolic equivalent cut points (METs) and multidimensional health target attainment were calculated [6]. Intensity thresholds were set using ubiquitous cut-points as follows (where 1 MET is equivalent to the basal metabolic rate (BMR) for each participant as calculated using the age and sex-matched Schofield equation [19]): Sedentary activity = <1.5 METs; Light activity = 1.5–2.9 METs; Moderate intensity activity = 3.0–5.9 METs; Vigorous intensity activity = 6.0–10.1 METs and Very vigorous intensity activity = ≥10.2 METs [6]. In order to complete the 7-day, 24-hour record, each minute of missing data where participants had removed the device as instructed was assigned that individual’s BMR [19].

Participants were invited to a digitally-recorded two-hour one-to-one interview conducted by the lead researcher (MW). Interviews primarily took place at the University of Bath (patients) or their place of work (HCPs). Participants were typically interviewed within 2–3 weeks of their physical activity monitoring period. The interview topic guides for HCPs and patients were compiled with input from an expert panel of academics and health professionals including 3 senior health psychologists, 2 senior health physiologists, 2 social marketers, a general practitioner and a research nurse. They included questions to capture interviewees’ views on physical activity and the importance they place on it (prior to seeing feedback), the preferences and comprehension towards the various feedback designs and the impact of receiving personalised physical activity feedback in terms of its motivational properties and practical application. Aside from the interpretation of their own feedback, HCPs were questioned about anticipated understanding from their patient’s perspectives (rather than themselves). Participants were shown the designs in a random order so that preferences were not influenced by exposure order. Each section of graphics and individual designs was given a brief verbal introduction by the interviewer.

### Analysis

Audio recordings were transcribed verbatim in Microsoft Word and then uploaded to NVivo (Version 9.0, QSR, Southport, UK) for coding and data organisation. The principles of Framework Analysis were used to analyse the data [20]. A period of familiarisation with the dataset by the lead researcher was followed by a process of coding whereby *a priori* themes directed by the interview topic guide, unexpected emergent themes and recurring viewpoints were identified. The accuracy of the initial themes, derived from a subset of the data, was confirmed by other members of the research team, and then used to guide the indexing of the remaining transcripts. The coding process enabled the development of lower order themes to be charted and organised into salient higher order themes that manifest within the whole dataset. At the final stage of data analysis, the derived themes for both groups were compared and similarities and differences were identified.

## Results

### Participants

We successfully recruited 30 patients and 15 HCPs who showed a diverse range of physical activity status. Of patients, 34% would have been considered sedentary, 45% moderately active and 21% highly active based on their total daily energy expenditure (based on a PAL of 1.40–1.69, 1.70–1.99 and 2.00–2.40, respectively). Similarly, 34% of HCPs would have been classified as sedentary, 53% moderately active and 13% highly active. One patient failed to complete the activity monitoring leaving 29 for analysis in that group. All other demographic and anthropometric characteristics of the study participants can be found in Table 1.

**Table 1:** Demographic characteristics of all participants included in the analyses.

Characteristic	Patient (n = 29)	HCP (n = 15)
Sex		
Male	21 (72%)	6 (40%)
Female	8 (28%)	9 (60%)
Age^a^	63 (7)	48 (10)
<45	1 (3%)	4 (27%)
45–54	2 (7%)	6 (40%)
55–64	9 (31%)	4 (27%)
65–74	17 (59%)	1 (7%)
Marital status		
Single	2 (7%)	3 (20%)
Married/ Civil partnership/ Cohabiting	22 (76%)	7 (47%)
Divorced/ Separated/ Widowed	5 (17%)	5 (33%)
Highest educational attainment		
None	2 (7%)	0 (0%)
GCSE or equivalent	7 (24%)	3 (20%)
A-Level or equivalent	3 (10%)	3 (20%)
1^st^ Degree or equivalent	12 (41%)	5 (33%)
Higher degree	5 (17%)	4 (27%)
Smoker		
Yes	2 (7%)	0 (0%)
No	27 (93%)	15 (100%)
Height (m)^a^	1.74 (0.10)	1.73 (0.09)
Weight (kg)^a^	82.0 (16.7)	76.7 (10.4)
BMI (kg/m^2^)^a^	26.9 (4.3)	25.7 (3.5)
Waist circumference (cm)^a^	95.0 (12.6)	84.5 (10.4)
Physical activity dimensions^b^		
Physical activity level^a^	1.83 (0.31)	1.72 (0.21)
Daily sedentary time (% waking day)^a^	68 (11)	69 (11)
Daily moderate activity (min/day)^a^	134 (75)	107 (45)
Weekly moderate-vigorous bouts (min/week)^a^	479 (361)	341 (208)
Weekly vigorous activity (min/week)^a^	100 (147)	125 (128)

^a^ = Values reported as mean (standard deviation)

^b^ = Physical activity dimensions that were presented in the ‘health target’ section of the feedback were as follows:
Physical activity level (PAL) was the average total daily energy expenditure/basal metabolic rate (Kcal/day); Daily sedentary time was the percentage of a 16 hour waking day (8 hours of sleep was assumed and subtracted from the total sedentary time) spent sedentary (<1.5 METs); Daily moderate activity was the average number of single minutes of moderate activity (≥3 METs, <6 METs); Weekly moderate-vigorous bouts included all activity greater than 3 METs sustained for at least a period of 10 minutes; Weekly vigorous activity combined all the minutes of vigorous activity (>6 METs) accumulated over the monitored week.

### Higher and lower order themes

**Fig 2:**
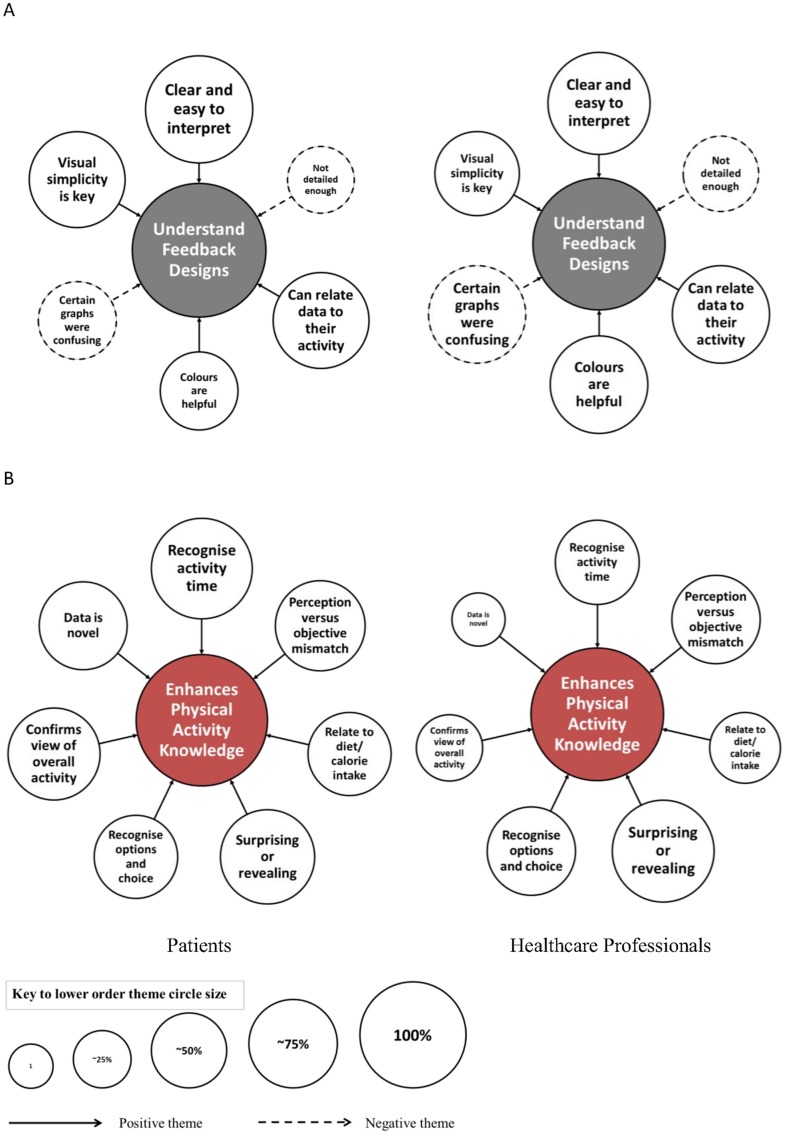
Component 1: Interpretation of the personalised feedback designs and data. Two higher order themes, represented by the large central circles, included the ability to accurately understand the visual physical activity data (A) and the enhancement of physical activity knowledge (B). The magnitude of the peripheral circles representing the lower order themes supporting the central theme, relate to the proportion of participants within each group identifying with each theme as indicated by the key at the foot of the figure.

**Fig 3:**
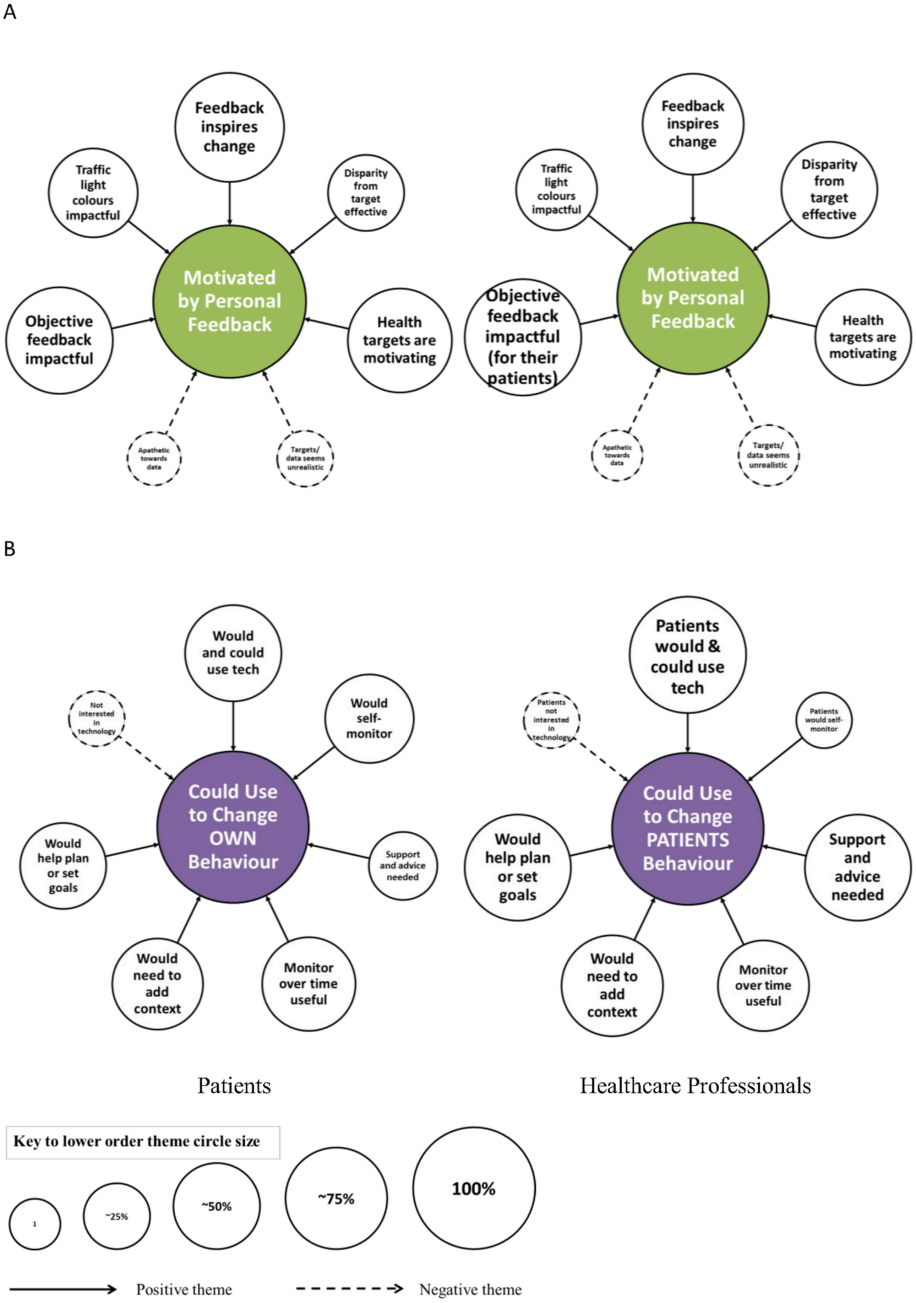
Component 2: The impact of personalised visual physical activity feedback on facilitation of health behaviour change. Two higher order themes (inner circles) included the motivation to change physical activity behaviour (A) and the usefulness of the personalised visual feedback to support health behaviour change (B). The magnitude of the peripheral circles representing the lower order themes supporting the central theme, relate to the proportion of participants identifying with each theme as shown by the key at the foot of the figure.

The analytical framework included two key components, the interpretation of the physical activity feedback designs and data (Fig 2), and the impact of personalised visual physical activity feedback on facilitation of health behaviour change (Fig 3). Indexing of lower order themes (peripheral circles) led to the emergence of two congruent higher order themes (inner circle) within each component of the framework. The lower order themes identified in the data that support these interpretations are quantified according to the number of respondents who shared that particular view. Lower order themes included in Figs 2 and 3 represent those that were identified in both patients and HCP groups. Additional lower order themes that were solely represented in one of the participant groups and example quotation extracts of the raw transcripts can be found in the supporting table (S1 Table). Where views within a group are contrasting, the opposing perspective was presented as a distinct theme (e.g. ‘handle and use technology’ and ‘dislikes technology’).

### Component 1—Interpretation of the personalised feedback designs and data

The higher order themes identified within the data included the ability of HCPs and patients to understand the comprehensive multidimensional feedback and the enhancement of their physical activity knowledge (Component 1, Fig 2). Similar proportions of HCPs (93%) and patients (100%) championed the clarity of certain visual images and were unified in their views on some of the more specific features such as the colours and simplicity of the designs. Only a very few participants felt that the images were not sufficiently detailed and 83% and 88% of patients and HCPs were able to easily relate the feedback to their behaviour in a meaningful way. Within the second higher order theme, a greater proportion of patients (72% vs. 20% for HCPs) felt that the data provided them with new information whilst more than 65% of both groups were able to recognise and accept the multidimensional nature of physical activity. Both groups were able to identify the times during their monitored week in which they were active at certain intensities and a large proportion of participants found aspects of their own personal feedback surprising, revealing or misaligned to their initial perception.

### Component 2—The impact of personalised visual physical activity feedback on facilitation of health behaviour change

The two higher order themes characterised by the analysis within the second component included the motivation to change physical activity behaviour and the usefulness of the personalised visual feedback to support health behaviour change (Component 2, Fig 3). Many of the lower order themes alluding to the positive motivational properties of the personalised feedback were evident in similar relative proportions of patients and HCPs. For example, 83% and 73% respectively found the feedback inspiring compared to only 7% of each group who demonstrated apathy towards the information. The health target data and the use of traffic light colours were acknowledged as key factors motivating individuals to want to increase their physical activity. A key discrepancy between the HCP and patient groups was their belief on the ability of patients to self-monitor their behaviour using the personalised feedback (13% vs. 55%) and on the need for additional support and guidance (80% vs. 28%). The two user groups were, however, more unified in their views on the utility of using technology to manage the feedback, plan and set goals, and the need to ensure the data was available longitudinally rather than as a simple snapshot.

## Discussion

We developed a promising and innovative way to present sophisticated physical activity profiles and feedback across key biologically healthful physical activity dimensions. Patients at risk of chronic disease and healthcare professionals who work with such patients expressed a clear ability to interpret the information and it was not perceived to be complex or confusing. The personalised feedback enhanced physical activity knowledge, was motivating and was reported to be a potential aide to the self-management of physical activity.

Physical activity has a critical role in the prevention of non-communicable disease [1] but translating this evidence into action has been challenging [21]. We have previously proposed that traditional conceptually-narrow approaches to physical activity do not provide individuals with sufficient information about the important aspects of behaviour, nor do they necessarily enable an individual to find tailored physical solutions that align with their interests and needs and are sustainable [6]. With technological innovation now already widespread, we are no longer constrained and can provide a much richer, more sophisticated and personalised profile regarding physical activity. In the present study, we demonstrate that patients value technology-enabled feedback about their activity and can grasp the innovative multidimensional portrayal of their physical activity. This gives encouragement that this sophisticated format of feedback is conceptually attainable for this population and that healthcare providers can trust individuals to handle more comprehensive physical activity information as this becomes increasingly accessible.

Participants in the present study also acknowledged an enhanced understanding of their own physical activity in response to receiving personalised feedback. Overall, a large proportion of participants found aspects of their own feedback surprising or revealing and demonstrated a misalignment between their perceptions and the objective data. A better understanding of their current physical activity could help individuals identify their relative strengths and shortcomings, make more informed decisions on how they might improve and set realistic goals [22]. For many participants the detailed minute-by-minute physical activity patterns helped them identify their activity and inactivity time, which could usefully be applied as a tool to communicate how even small changes can be important for reducing health risk [23]. Encouragement can also be taken from the recognition of the options and choices in their multidimensional profiles, which, as an approach to the presentation of meaningful feedback, would offer patients the chance to find sustainable solutions aligned to their personal preferences and needs.

The provision of bespoke options and heightened awareness may provide individuals with a sense of attainable and volitional solutions rather than prescribed choice which, in turn, is likely to improve the quality of their motivation and prolonged engagement in physical activity [24]. A large proportion of individuals in the present study highlighted the multidimensional health targets, the use of a comparative discrepancy between target and performance and the traffic light colours as factors that inspired them to contemplate change. This alleviates fears that multidimensional feedback might be complex and/or confusing and, whilst the assertions made by the patients and HCPs about their desire to change are prospective, our results suggest that this approach may be a useful motivational resource if applied appropriately.

Many theoretical frameworks applaud the role of feedback, self-monitoring and goal-setting as key constituents for successful and sustained lifestyle modifications [2,3,25]. However the challenge to date has been finding the most effective way of implementing such strategies [26]. Interestingly, in the present study, a large proportion of patients felt that they could effectively self-monitor their own physical activity behaviour without additional support using the presented feedback and expressed confidence in using technological platforms to do so. HCPs on the other hand were somewhat sceptical of patients’ ability to self-monitor in the absence of any support and guidance. Speculatively, this contrasting view may be reflective of a greater wealth of experience that HCPs have with patients acting on their advice and/or the challenges associated with setting realistic goals, adhering to lifestyle modifications and sustaining behaviour change. Nonetheless, the optimism and enthusiasm of patients to use the feedback presented here suggests that this offers a promising strategy for supporting behaviour change. These findings are useful to researchers who are interested in capitalising on technological innovation to provide physical activity feedback across various biologically important and healthful physical activity dimensions. Prior research indicates that the effectiveness of technology-enabled health behaviour interventions is likely to be enhanced when the patient is involved in its development [27,28] and particularly in the application of physical activity feedback [29,30]. In this regard, we have used these results to inform a randomised controlled trial (Mi-PACT, ISRCTN18008011) that is currently underway and that will determine whether the provision of multidimensional personalised feedback helps patients to change their physical activity and reduce risk of chronic disease.

## Conclusions

In conclusion, using appropriate graphics and visualisations, multidimensional and sophisticated physical activity feedback can be presented to patients in a way that is informative and understandable rather than complex and confusing. For the first time, we show that a targeted clinical population can accurately interpret comprehensive multidimensional physical activity information and that this information is potentially motivating for this population. As technology for monitoring physical activity becomes more accurate and affordable, we can move beyond simple physical activity messages and there is an exciting opportunity to generate an integrated and holistic picture of physical activity that is more informative and tailored to an individual’s needs, preferences and abilities.

## Supporting Information

S1 Fig Example physical activity profile portfolio for an individual including all nine feedback graphics shown to participants. Participants were given a short introduction to each section within the interview and then shown and asked to comment on each depiction of their feedback in turn. Graphics were shown in a random order per section and participants were given the key to intensity thresholds on page 4 for reference whilst interpreting graphs A to F.
(PDF)

S1 Table Extracts of raw data sources used to exemplify lower themes identified under the two components of the Framework analysis. Identified themes are in a clockwise order that they appear in Figs 2 and 3 within the main text and are accompanied by a quote and the percentage (%) of participants in which the theme was identified. Lower order themes under the dotted lines represent single items not included in the figures and represent those lower order themes that were solely identified in one of the participant groups (i.e. only patients or healthcare professionals) for each higher order theme.
(DOCX)
