# Supplementary material for: The Understanding and Interpretation of Innovative Technology-Enabled Multidimensional Physical Activity Feedback in Patients at Risk of Future Chronic Disease
Source: PLoS One. 2015 May 4;10(5):e0126156. doi: 10.1371/journal.pone.0126156 (PMC4418766; doi:10.1371/journal.pone.0126156)

Department for Health | University of Bath

MiPACT PROJECT

Physical Activity Profile Portfolio

Table of Contents

Section 1: Energy Expenditure 3

Key to Intensity Thresholds 4

A: 24 Hour Bar 5

B: 24 Hour Radial 6

C: Weekly Data 7

Section 2: Summary Data 8

D: Bubble 9

E: Bar 10

F: Pie 11

Section 3: Health Targets 12

G: Wheel Of Activity 13

H: Target Bars 14

I: Sliding Targets 15

We have measured your 24-h daily energy expenditure (i.e. the number of calories you burn per day)

For each day we have collected 1440 minutes of data!

There are a variety of ways of presenting such a large amount of information.

In this section, we will present your individual data (for one 24-h day) in a number of different ways.

After a brief introduction to each graphic we will ask you a few questions regarding your thoughts, opinions and preferences.

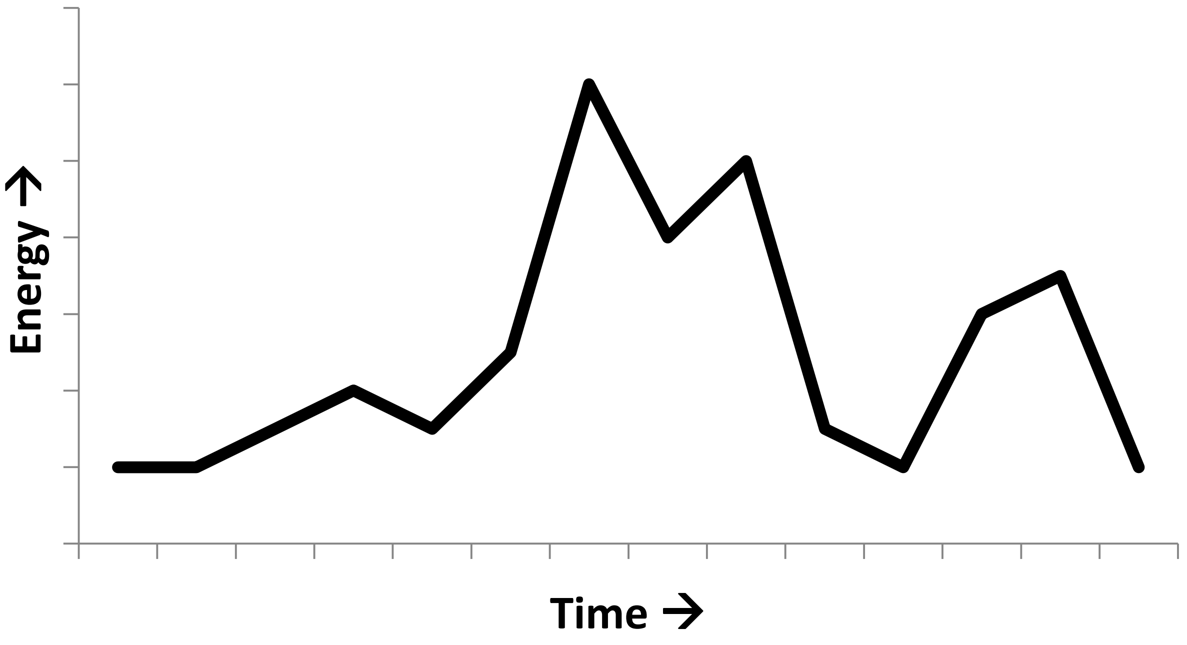

Key to Intensity Thresholds

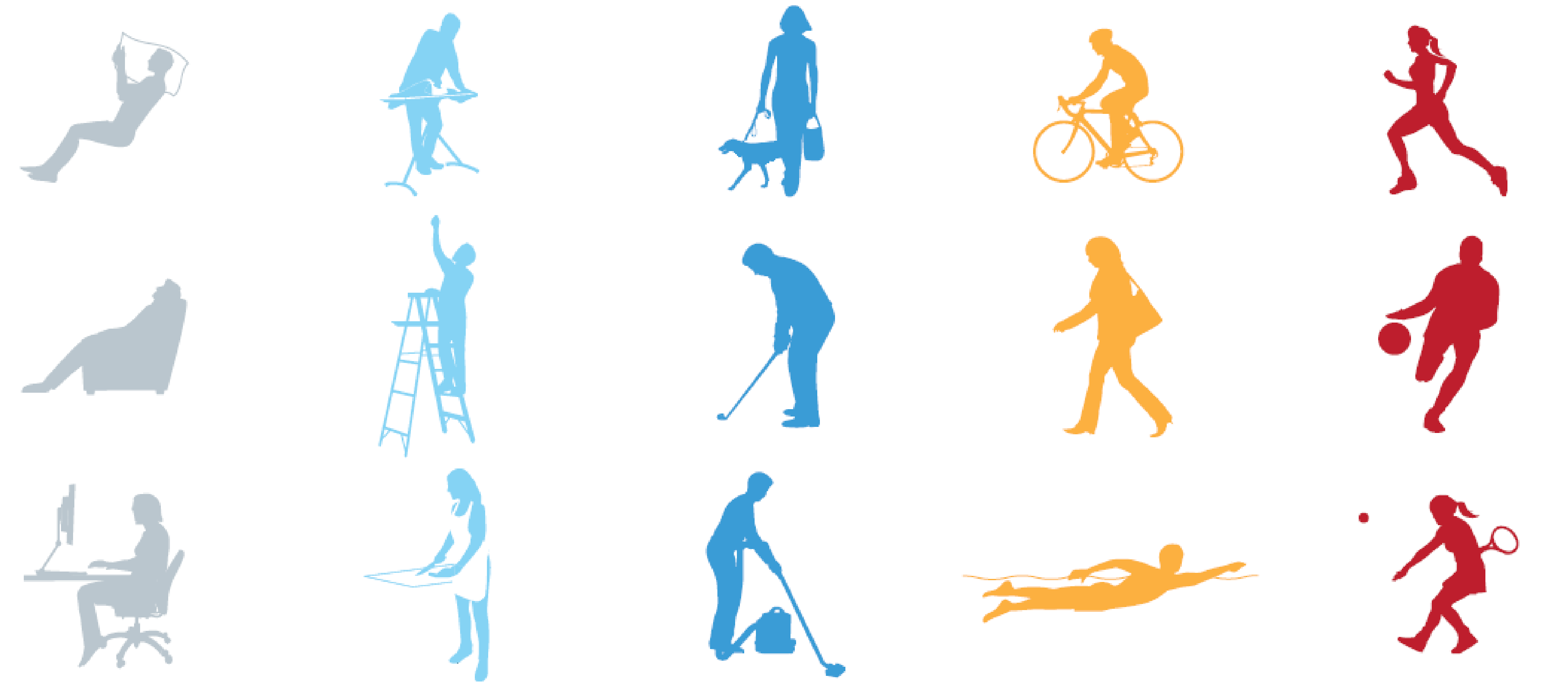

Sedentary: Sitting and/or lying (reading, TV, video games, talking), computer work (desk based or seated)

Light: Light home-based activities (food preparation, washing dishes, ironing, light cleaning)

Moderate: Walking (with dog, shopping), golf, moderate intensity home and garden (hoovering, sweeping, mowing lawn)

Vigorous: Brisk walking (flat or uphill), cycling, swimming and jogging

Very vigorous: High intensity competitive sports, running, squash and basketball

A – 24 Hour Bar

Sedentary
Light
Moderate
Vigorous
Very Vigorous

Saturday 23/03/2013
Total Calories: 2614

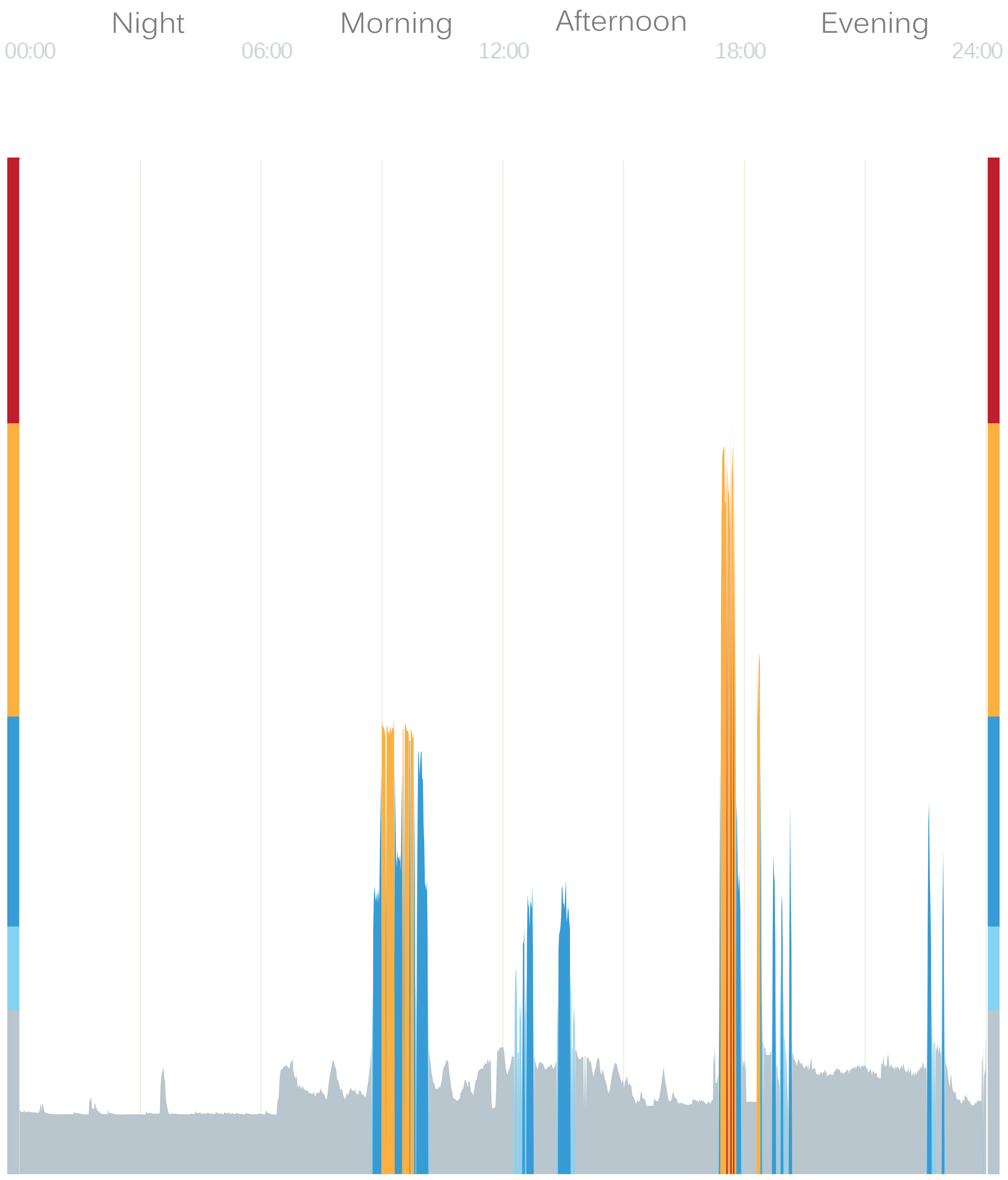

B – 24 hour Radial

Saturday 23/03/2013
Total Calories: 2614

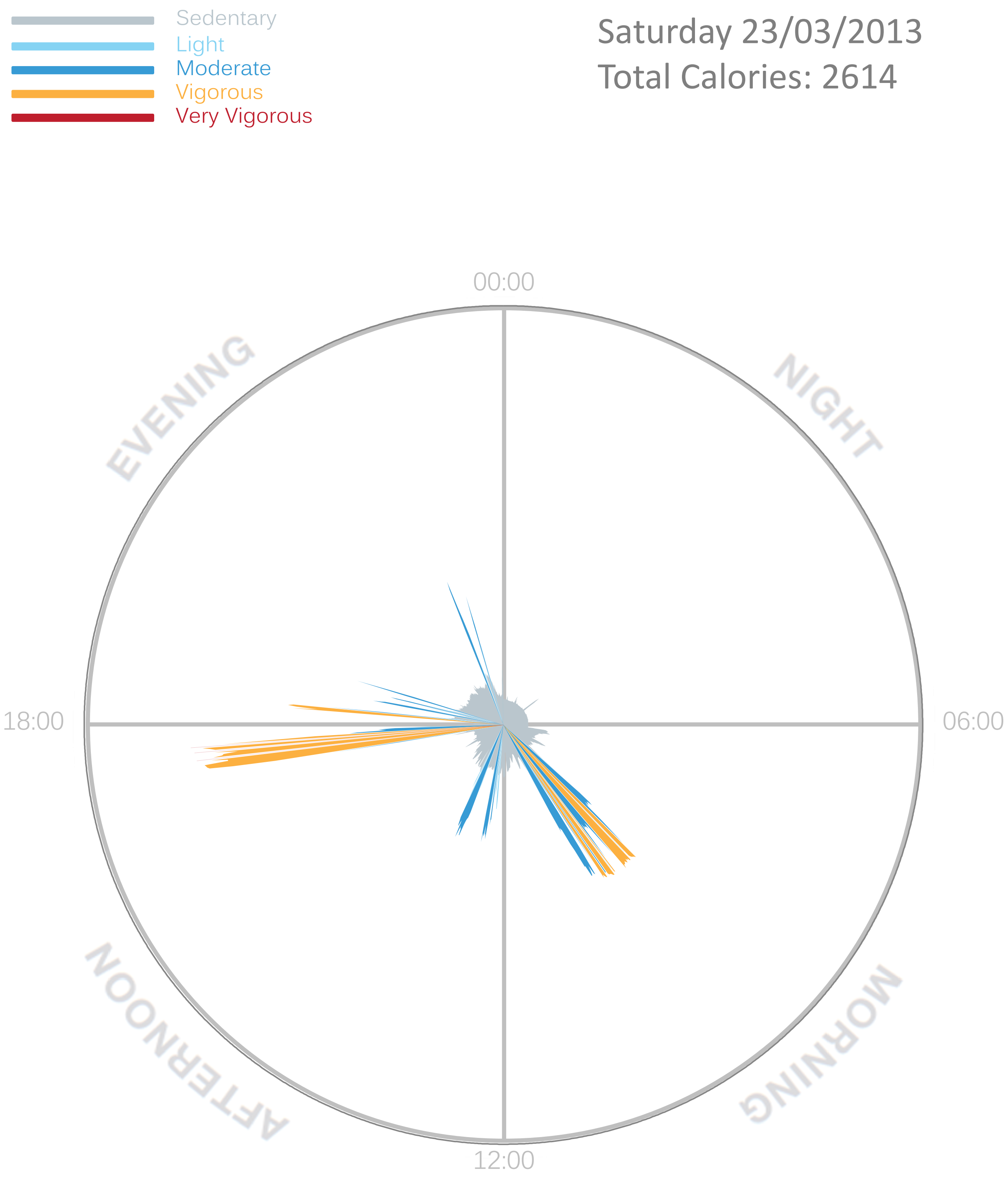

C – 7 Day Data

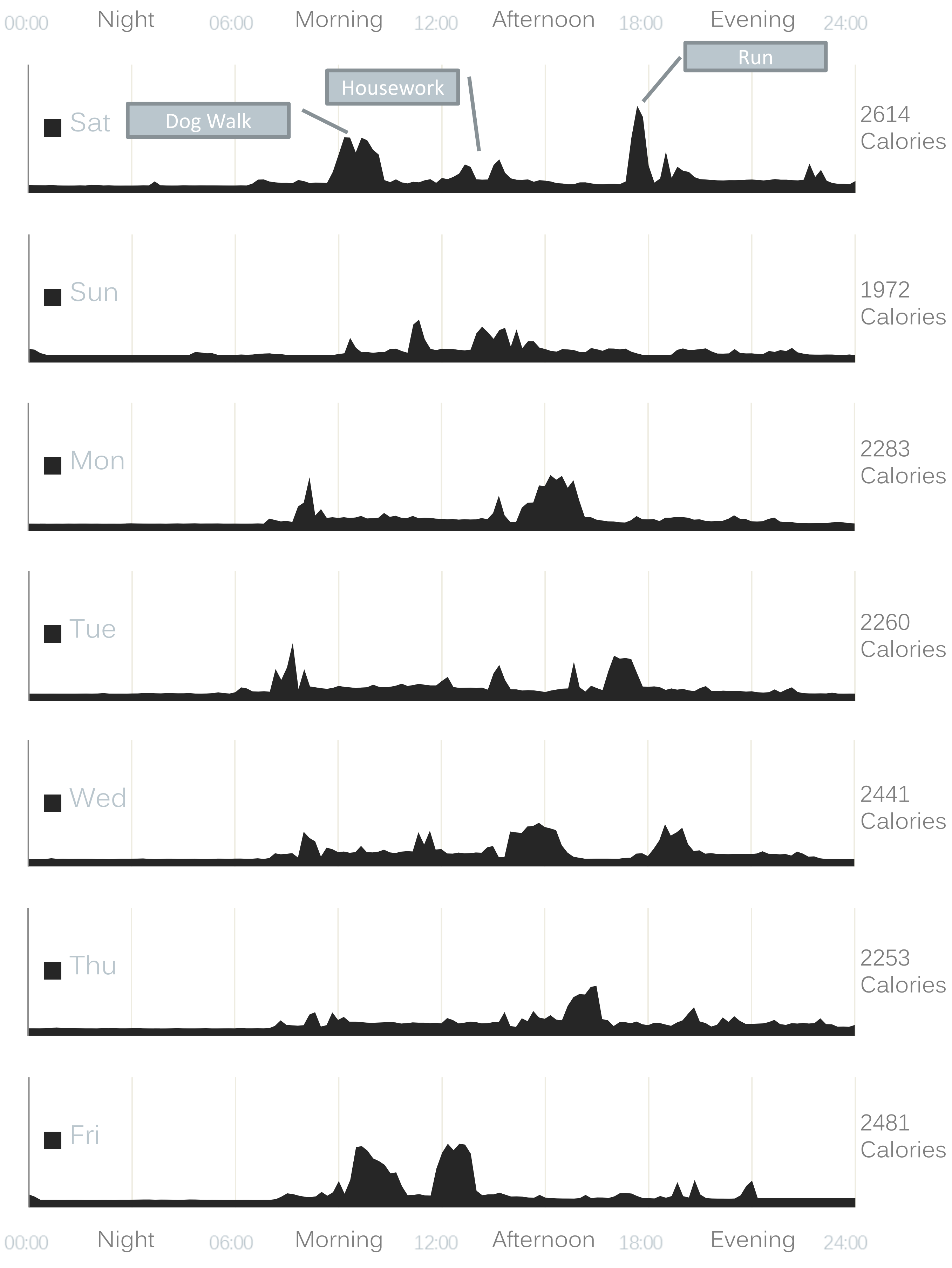
00:00
Night
06:00
Morning
12:00
Afternoon
18:00
Evening
24:00
Dog Walk
Housework
Run
Sat
2614 Calories
Sun
1972 Calories
Mon
2283 Calories
Tue
2260 Calories
Wed
2441 Calories
Thu
2253 Calories
Fri
2481 Calories
00:00
Night
06:00
Morning
12:00
Afternoon
18:00
Evening
24:00

We have shown you various ways of displaying your daily or weekly activity patterns, we can now pick out key summary information.

For example we can display the average and total time spent in each activity intensity threshold during your week.

We can also summarise the amount of calories expended at each of these intensity thresholds.

The table below is used to describe the relationship between time and energy within each activity threshold.

You will now be shown some visual images of your summary data.

Activity Intensity	Time (Minutes)	Calories	Calories	Time (Minutes)
Sleep	30	35	500	400
Sedentary	30	50	500	275
Light	30	100	500	150
Moderate	30	180	500	80
Vigorous	30	300	500	50
Very Vigorous	30	425	500	35

D – Bubble

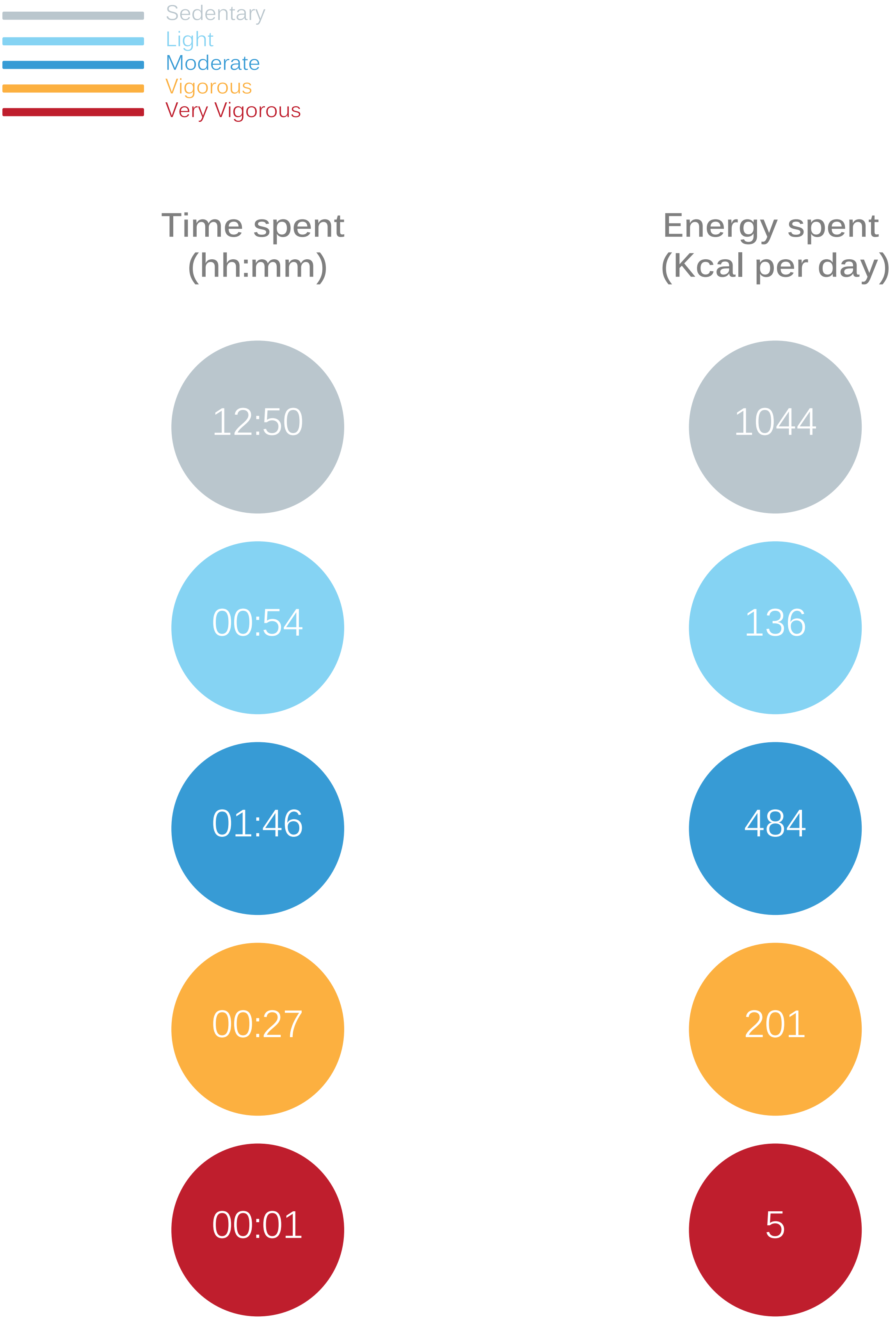
Sedentary
Light
Moderate
Vigorous
Very Vigorous
Time spent (hh:mm)
Energy spent (Kcal per day)
12:50
1044
00:54
136
01:46
484
00:27
201
00:01
5

E – Bars

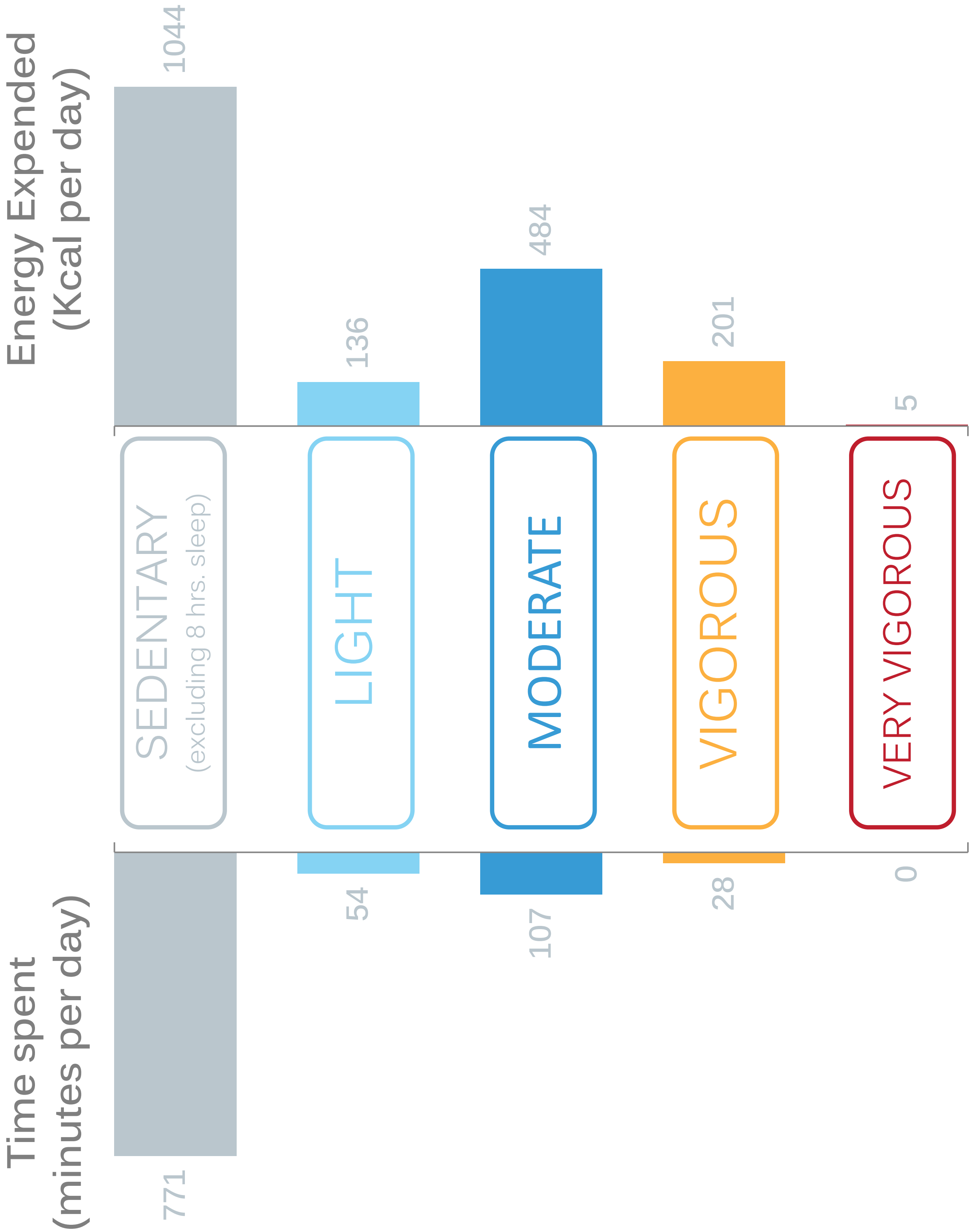

Energy Expended
(Kcal per day)
1044
136
484
201
5
SEDENTARY
(excluding 8 hrs. sleep)
LIGHT
MODERATE
VIGOROUS
VERY VIGOROUS
771
54
107
28
0
Time spent
(minutes per day)

F – Pie

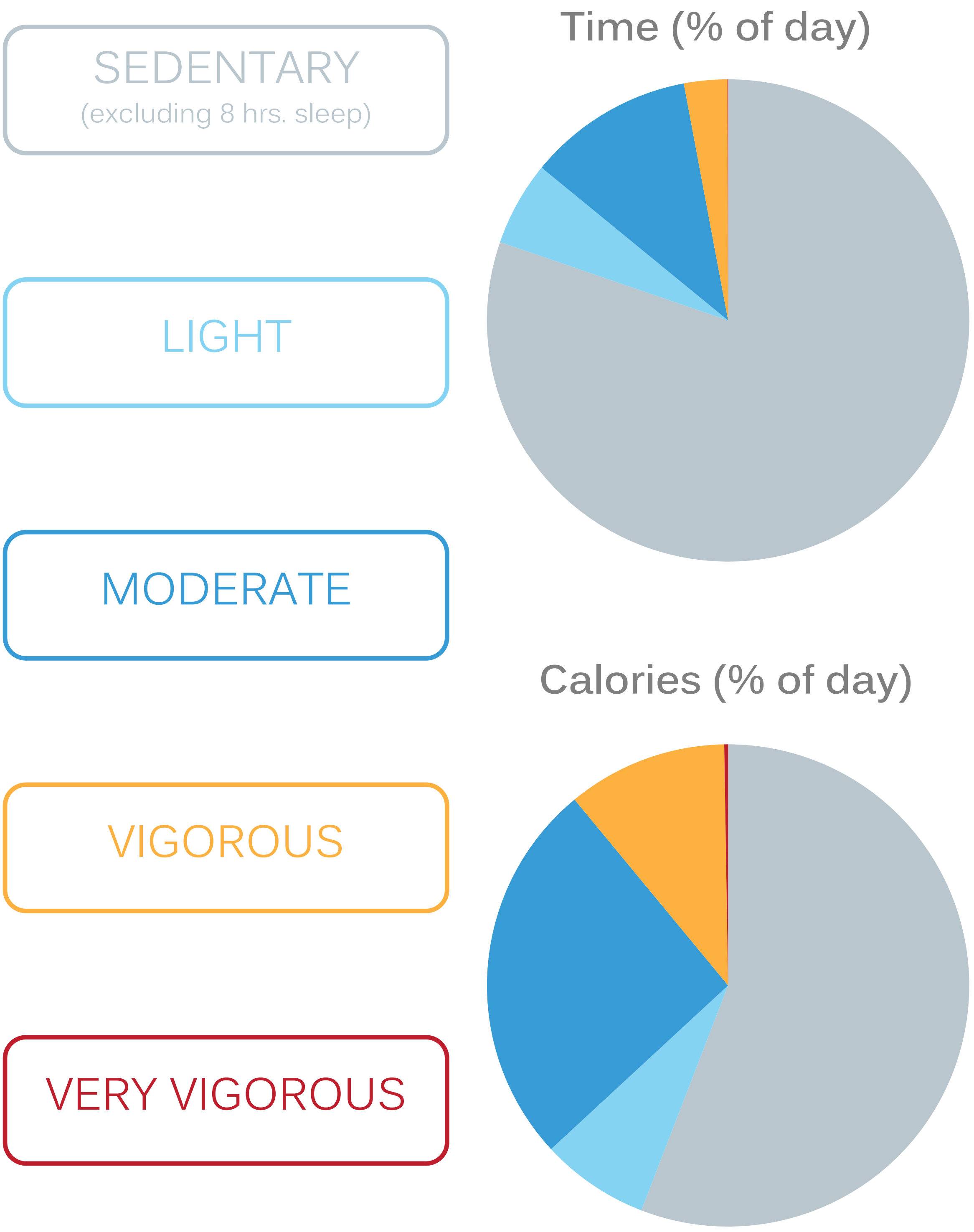
SEDENTARY
(excluding 8 hrs. sleep)
LIGHT
MODERATE
VIGOROUS
VERY VIGOROUS
Time (% of day)
Calories (% of day)

Section 3 – Health Targets

Further to summarising your activity data, we can now show how this sits with current health recommendations.

These recommendations are set based on levels of activity associated with risk for a variety of health problems.

Here we present 5 physical activity targets which have independent effects on your health risk.

There are therefore various aspects of your physical activity profile that can be altered to improve your health.

The 5 dimensions are:

- **Daily calorie burn**: PAL ≥ 1.75
- **Weekly moderate activity**: 120 accumulated minutes
- **Moderate 10 minute bouts**: 150 minutes per week
- **Vigorous activity minutes**: 75 minutes per week
- **Sedentary time**: < 60% of waking day

This section will use a traffic light colour system to indicate whether you are under, near or over the target.

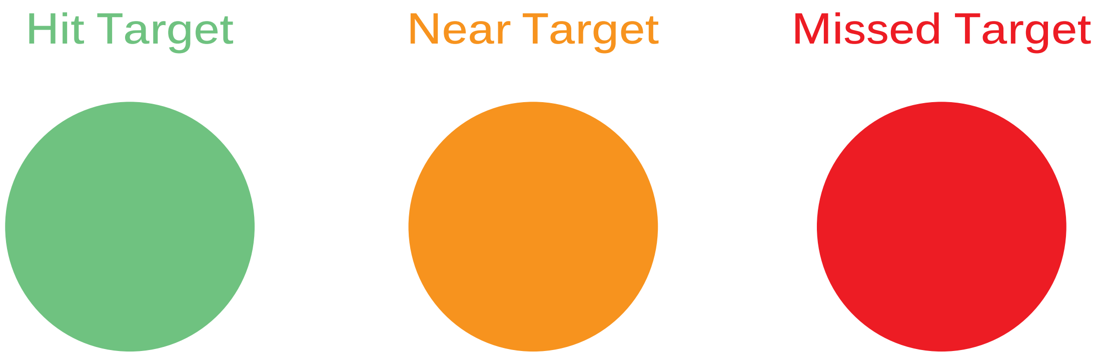

G – Wheel of Activity

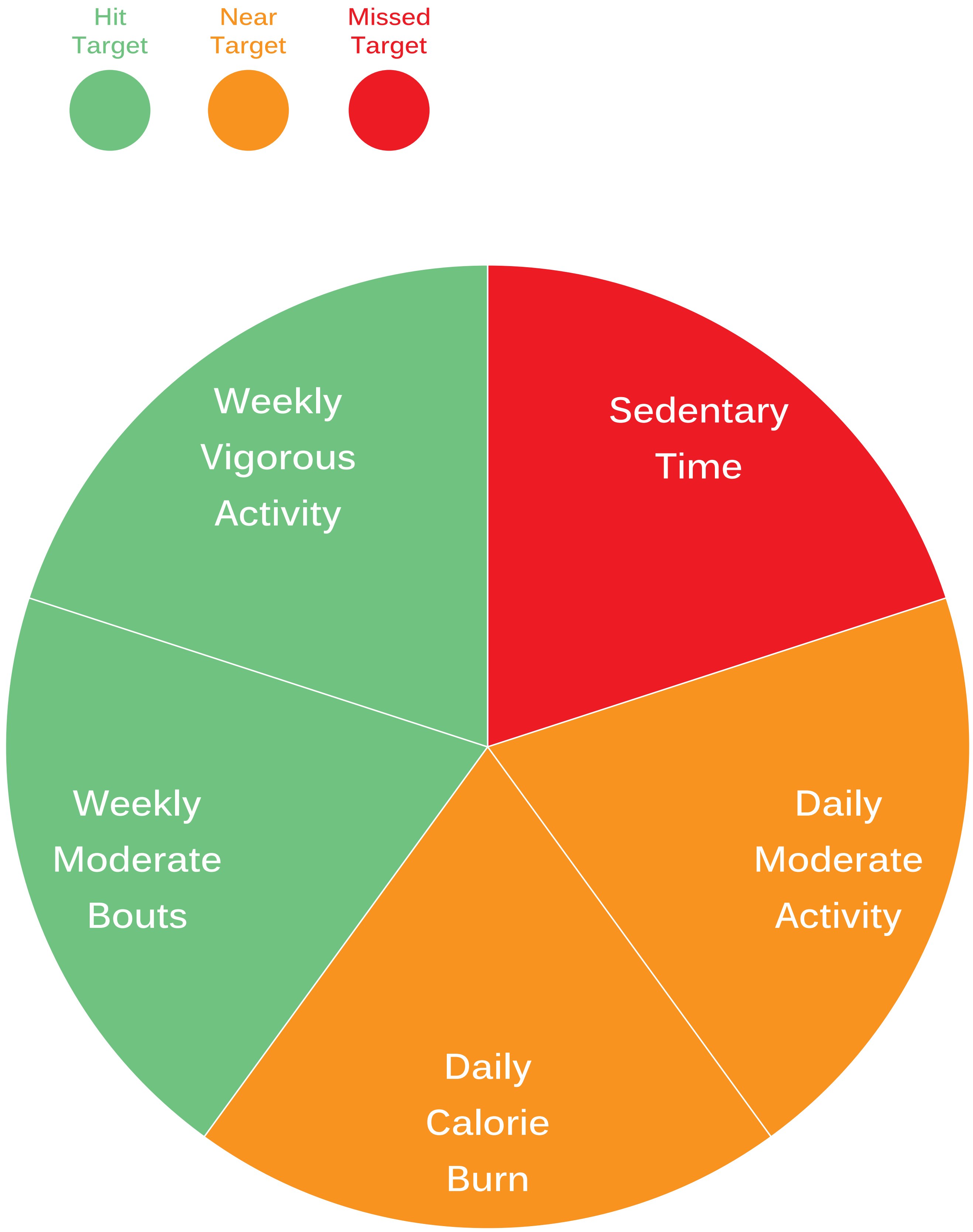

H – Target Bars

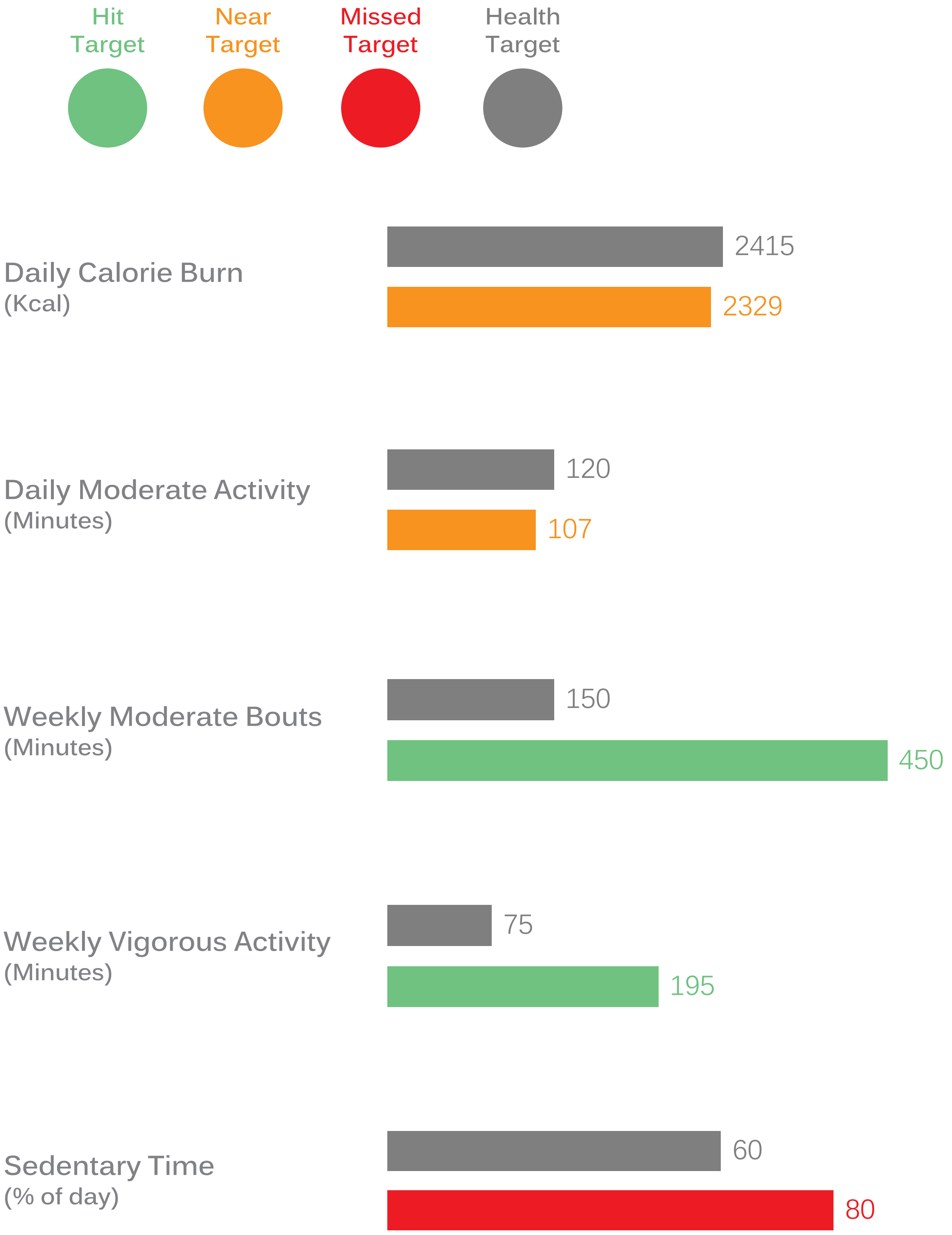

I – Sliding Targets

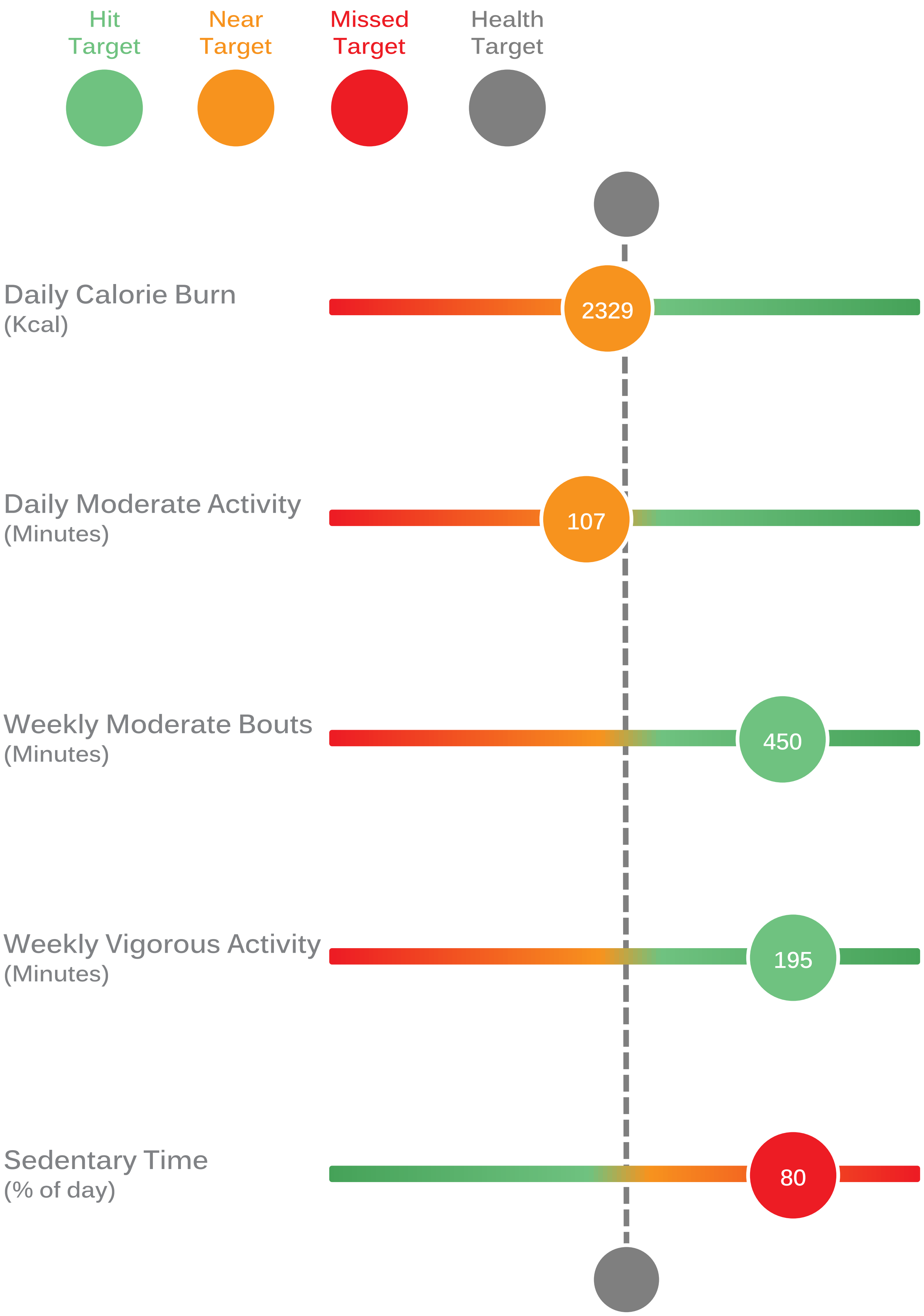

Supplement: S1 Fig — Participants were given a short introduction to each section within the interview and then shown and asked to comment on each depiction of their feedback in turn. Graphics were shown in a random order per section and participants were given the key to intensity thresholds on page 4 for reference whilst interpreting graphs A to F. (PDF) [file pone.0126156.s001.pdf]
