# Supplementary material for: The Understanding and Interpretation of Innovative Technology-Enabled Multidimensional Physical Activity Feedback in Patients at Risk of Future Chronic Disease
Source: PLoS One. 2015 May 4;10(5):e0126156. doi: 10.1371/journal.pone.0126156 (PMC4418766; doi:10.1371/journal.pone.0126156)
Supplement: S1 Table — Identified themes are in a clockwise order that they appear in Figs 2 and 3 within the main text and are accompanied by a quote and the percentage (%) of participants in which the theme was identified. Lower order themes under the dotted lines represent single items not included in the figures and represent those lower order themes that were solely identified in one of the participant groups (i.e. only patients or healthcare professionals) for each higher order theme. (DOCX) [file pone.0126156.s002.docx]

| Lower Order Theme | **Evidence (Quotations)** | | | |
| --- | --- | --- | --- | --- |
|  | **Patient (29)** | **%** | **Healthcare Professional (15)** | **%** |
| **Component 1: Interpretation of the feedback designs and data** | | | | |
| **Higher Order Theme: Understand Feedback Designs** | | | | |
| Clear and easy to interpret | *…Yeah well that’s quite interesting just...I can clearly see which days I do activity, that’s obviously more activity throughout the day…* | 100 | *…Yeah I think for someone who is um, not doing any exercise at all that would be enough really, yeah definitely, that to me would be very simple for them to see. It’s very clear….* | 93 |
| Not detailed enough | *…No I don’t find that particularly helpful um, once you’ve analysed this one and this one that doesn’t really add anything to it, not to my mind…* | 38 | *…Um I guess the problem with that is it just shows me bad, but it doesn’t really tell me how much I need to change to make better….* | 53 |
| Can relate data to their activity | *… Remember that day yes I was playing in Bristol, kind of a long day. Gardening. That would probably have been travelling back from golf I suppose. It’s interesting that Pilates doesn’t spike up more…* | 83 | *…that’s why the temperatures and the calorie expenditure would be high in the evening. So it’s gym there and then, running classes there… and similarly here…Thursday with the circus as well…* | 87 |
| Colours are helpful | *…Well again I think the uh…the colour is going through all this you get to know what the colours represent so it makes it easier to read together…* | 66 | *…Right that’s really interesting and that is clear now because the colours the colours make that clear…* | 87 |
| Certain graphics were confusing | *…there’s a slight confusion in my mind I suppose because that is… but that is calories, you know the units change that’s minutes that’s percentage...but you only have to read it to understand it…* | 55 | *…* *The ‘E’ bar…it’s not clear…without spending time looking at it and analysing it. Whereas that, that’s quite clear isn’t it? Just by looking at it simply…* | 87 |
| Visual simplicity is key | *…It’s just simpler it tells me exactly the same I can see my performance against the recommending one and it’s an easy comparison there, each of the categories and it’s nice and simple…* | 83 | *…I just like this one here because I think it’s very clear very visual very simple, and it’s straight to the point…* | 67 |
| Used to Seeing Graphics | *…Keep it as plain as you possibly can and as simple as you can. I used to do lots of presentations with charts and things and I know simple, people understand…* | 48 | *-* | - |
| Confused by multiple targets | *-* | - | *…But maybe these targets, when you start presenting them together, it’s almost like there’s inconsistency between them. Yeah, so that’s the first impression…* | 20 |
| **Higher Order Theme: Enhances Physical Activity Knowledge** | | | | |
| Recognise activity time | *…Yeah yes. Especially when you can identify the exact time that that represents, as I say you can actually break it down into what it was that caused that spike. Excellent…* | 93 | *..Moderate mostly in the morning, lunchtime, and then….I don’t know it sort of fades out, very little in the evening. Very little vigorous exercise in the evening. Mainly moderate. And good night’s sleep...* | 67 |
| Perception versus objective mismatch | *…Yeah I am surprised that that to be honest with you the sedentary yeah, there is more there than, than I thought to be honest…* | 76 | *…yeah so I thought it’d be a lot higher than that. From what I think is vigorous activity I thought it’d been...but like you said it’s all right isn’t it…* | 73 |
| Relate to diet/ calorie intake | *…how you fill in that calorie gap with food. That’s the next part of it really I suppose. Presumable if you’re filling yourself up with food the balance would change wouldn’t it…* | 66 | *interesting to see how many calories you’ve used each day... in comparison to, well I know what sort of food intake I consume,* | 47 |
| Surprising or revealing | *…I'm sort of, I'm surprised by the results really because although I feel healthy, and I eat well, I'm surprised that I'm not sort of just this side of the line. I would imagine that I am a bit too sedentary really for, for health but hey…* | 83 | *…I’m surprised I haven’t ever reached the category of very vigorous because sometimes when I’ve done a hard step class or something I think I’ve worked really hard, I’m quite…that surprised me, has surprised me…* | 93 |
| Recognise options and choice | *…Yeah it does. Because doing one would sort these two so...um, that would be my aim is to work on those two really. By the nature of it that would bring that one down wouldn’t it…?* | 66 | *…I like this idea that you say that you target one section, one segment, and um…and I think it’s a really good way of letting them work on something…* | 73 |
| Confirms view of overall activity | *…Yes in that it confirms what I already knew to a point. Yeah it’s just nice to see it in front of you what your average week is like. So yeah I’m fine with that…* | 79 | *…again it confirms the picture of an overall sedentary life with big bursts of energy here and there basically. Thank goodness I cycle, if I didn’t cycle id just become flat lined wouldn’t it…* | 40 |
| Data is novel | *…Very interesting yes. I wondered what it was all doing, I must say it is interesting…* | 21 | *…Well I’ll I don’t know how to respond really, I’ve never seen anything like it before, I’ve never seen my days portrayed like that…* | 3 |
| **Component 2: The impact of personalised visual physical activity feedback on facilitation of health behaviour change** | | | | |
| **Higher Order Theme: Motivated by Personal Feedback** | | | | |
| Feedback inspires change | *…seeing the data laid out makes me think I’m not as active as I should be and that I have to do more to maintain my health or to improve it if I possibly can…* | 83 | *…it makes me feel that I must try harder. Room for improvement, but, then that’s all of us…* | 73 |
| Discrepancy from target effective | *…I think that it's the length of the bar; you know you can graphically say ‘hey look you know my target is only there and I'm just short of it…* | 38 | *…It’d be nice to do that whole test again but this time...but then I suppose you’d try and achieve something more and see what you do but um, no it’s good…* | 67 |
| Health targets are motivating | *…the targets I think. Um, that I think has got me going more than any of the data. The others you can see where you are and what’s expected, when you see the targets it gives you incentive if I see I missed those targets how to meet them…* | 76 | *…I think it’s just encouraging to have it all in front of you, and then go through it, and um….it makes me want to do more, so I think it would make other people want to do more…* | 67 |
| Targets /data seems unrealistic | *…That's quite a lot actually as a target I'm comfortably achieving that at the moment but for somebody in a full-time job with commuting at either end of the day that's going to be really hard…* | 10 | *...it’s encouragement that matters I think. Rather than showing people how inactive they are. That’s not going to help them to do anything about it…* | 20 |
| Apathetic towards data | *…I think that’s the problem because mine’s all green obviously those don’t mean much…* | 7 | *…But, and that hasn’t, I’m not sure this will prompt me to do anything about it actually, uh because I thought I would be prompted to do more when I was wearing the monitors actually but it didn’t…* | 7 |
| Objective feedback is impactful (for patients) | *I think just seeing your actual results is good… anything preventative for national health has got to be good so if this is going to...I know it’s not a preventative thing but it’s to help me keep my health so it’s a way forward isn’t it? It’s showing me on my own things what I should be doing. Yeah no it’s been very helpful.* | 79 | *…but I think it really could make someone sit back and realize wow, just maybe an extra 10 minutes here or doing something like that. Not a massive change, but could really have a dramatic change on an overall week of what they do so yeah…* | 93 |
| Traffic light colours impactful | *…I’m concerned but clearly if one was orange or one was red it would stand out as an area I needed to do something about be it not sitting around so much or a bit more short bursts and things…* | 55 | *…Yeah I’m uh, a little bit disappointed. There’s such a big red ‘miss target’, um…but, I think if, well, I have seen it so, I will try and do something about it because it makes me feel bad…* | 47 |
| May put patients off | *-* | - | *…And its encouragement that matters I think. Rather than showing people how inactive they are. That’s not going to help them to do anything about it I don’t think…* | 20 |
| **Higher Order Theme: Could use to change own (patients) behaviour** | | | | |
| (Patients) would and could use tech | *…I’d love to and as I’ve said to you I’m sort of a silver surfer and modern technology is something that um, I don’t find easy but I keep sort of having battles with it and hoping I win. So I’d be happy to...I would be very interested…* | 66 | *…even though some of our older people might not have the technology – even though the ones I tend to deal with in our specialist groups all seem to have computers – all the family have them, or they’ve got smart phones…* | 93 |
| (Patients) would self-monitor | *…I’d want to have the monitor but then also have some way...what I’d like to do is have the monitor, and download the info onto my PC, I’d be able to take the monitor off, download the data at the end of the day, or at the end...do it myself, and I’d probably want to do it on a daily basis...* | 55 | *…I think most people would be able to manage it, yeah. Yeah, definitely you might get the odd one two perhaps, you know…but I think most people would…* | 13 |
| Support and advice needed | *…So um, that sort of information is what you would need to have available to support or whoever's going to be their follow-up support would need that sort of information…* | 28 | *…I think in people who aren’t already exercising I think they would need on-going support or prompting to continue doing something….* | 80 |
| Monitor over time useful | *…No absolutely. Yes it’d be interesting if you could know it every and compare every week as I say one month to another and one season to another...as a relatively short period of time it wasn’t necessarily a normal week …* | 62 | *…I’d want to say right okay give me 6 months to get my act together and let me come back again and see if I’ve actually improved and I think that would be of benefit…* | 60 |
| Would need to add context | *…could give a quick suggestion on ways you could change that pattern to your benefit and that would be easy to use as a basis I would’ve thought. You spend an extra 20 minutes a day on vigorous exercise you’re going to increase that a lot more than if you’re a sedentary person…* | 59 | *…I think your challenge would be just – or the challenge of the health care professional using your data – would be to turn that into alter their thoughts to if they’re doing well – so tell them what they’re doing well…* | 73 |
| Would help plan or set goals | *… or you can tell it, well I’ve got free evening there or a free afternoon there and it can suggest an activity that you can do that would get you up to the target. Yes I like that! Yeah something proactive yeah…* | 52 | *I think it would do because you know you’re asking somebody, ‘what are your goals’ …and if they’ve got nothing coming up, whereas here it could be ‘well actually yeah I would like to increase my calories a bit more so how are we going to do that?* | 80 |
| (Patients) Not interested in technology | *…No I'm one of the few I don't go on the computer a lot, no actually…* | 10 | *…I’m still amazed at what high percentages do not use the internet. And they’re scared of it and um, even if they do use it, there are an awful lot of people that are very limited in what they use it as…* | 13 |
| Feedback needs to be tailored | *-* | - | *…I mean it’s...everyone’s different isn’t it, how they portray something how they perceive it and how they understand it, everyone’s going to be different I think so, what I might suggest I mean I like that but some other person might come in and go ‘it doesn’t mean anything to me’…* | 87 |
